# Hypoxia-mimicked mitochondrial stress triggers APOBEC3A-mediated DNA damage via non-canonical innate immune activation

**DOI:** 10.1093/narmme/ugag012

**Published:** 2026-02-03

**Authors:** Rodolphe Suspène, Béibhinn O’Hora, Constance de Maere d’Aertrycke, Lydie Couturier, Théo Defresne, Pierre Khalfi, XiongXiong Li, Jean-Pierre Vartanian

**Affiliations:** Virus and Cellular Stress Unit, Department of Virology, Institut Pasteur, Université de Paris Cité, 28 rue du Dr. Roux, F-75724 Paris cedex 15, France; Virus and Cellular Stress Unit, Department of Virology, Institut Pasteur, Université de Paris Cité, 28 rue du Dr. Roux, F-75724 Paris cedex 15, France; Complexité du Vivant, Sorbonne Université, 75005 Paris, France; Virus and Cellular Stress Unit, Department of Virology, Institut Pasteur, Université de Paris Cité, 28 rue du Dr. Roux, F-75724 Paris cedex 15, France; Virus and Cellular Stress Unit, Department of Virology, Institut Pasteur, Université de Paris Cité, 28 rue du Dr. Roux, F-75724 Paris cedex 15, France; Virus and Cellular Stress Unit, Department of Virology, Institut Pasteur, Université de Paris Cité, 28 rue du Dr. Roux, F-75724 Paris cedex 15, France; Complexité du Vivant, Sorbonne Université, 75005 Paris, France; Virus and Cellular Stress Unit, Department of Virology, Institut Pasteur, Université de Paris Cité, 28 rue du Dr. Roux, F-75724 Paris cedex 15, France; Lanzhou Institute of Biological Products Co., Ltd (LIBP), subsidiary company of China National Biotec Group, Company Limited (CNBG), 730046 Lanzhou, China; Virus and Cellular Stress Unit, Department of Virology, Institut Pasteur, Université de Paris Cité, 28 rue du Dr. Roux, F-75724 Paris cedex 15, France

## Abstract

Hypoxia is a hallmark of the tumour microenvironment, driving metabolic reprogramming, immune activation, and genome instability. Here, we showed that cobalt chloride (CoCl_2_), a hypoxia-mimicking agent, potently induces the expression of the DNA cytidine deaminase *APOBEC3A (A3A)* in THP-1, a human monocytic cell line. *A3A* upregulation occurred in a dose-dependent manner, independently of type I interferon signalling, and was accompanied by increased double-strand DNA breaks. Transcriptomic profiling revealed broad hypoxia-driven reprogramming, characterized by activation of the stress response and downregulation of mitochondrial signalling pathways. Mechanistically, cobalt chloride induced mitochondrial dysfunction, metabolic reprogramming, and cytosolic release of mitochondrial DNA (mtDNA). Cytosolic mtDNA was transcribed by RNA polymerase III into immunostimulatory RNA, which activated the RIG-I/TRAF6/NF-κB signalling cascade to drive *A3A* expression. Inhibition or knockdown of RNA polymerase III markedly reduced both *A3A* levels and DNA damage, highlighting the central role of this pathway. All together, our findings reveal a novel interferon-independent signalling route through which hypoxia-induced mitochondrial stress activates A3A, directly linking metabolic dysfunction to genome instability. This mechanism involves mitochondrial perturbation as a key driver of APOBEC3-mediated mutagenesis in hypoxic tumours and other diseases associated with mitochondrial stress.

## Graphical Abstract

**Graphical Abstract:**
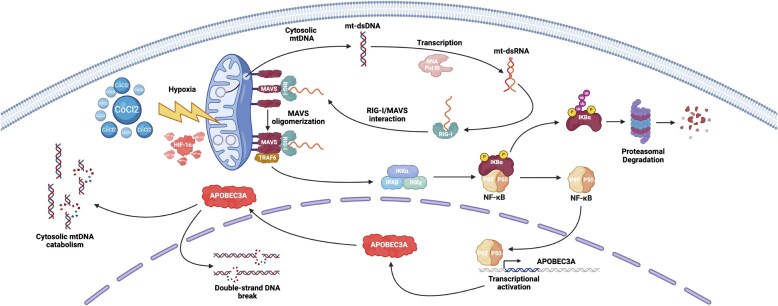

## Introduction

Cellular stress is a defining feature of the tumour microenvironment and is intimately linked to the initiation and progression of cancer [1]. Tumour cells are subjected to a wide range of stresses, both intrinsic and extrinsic, including oxidative and genotoxic stress, hypoxia, or nutrient deprivation [2]. These stress conditions challenge cellular homeostasis and activate various adaptive responses. While such responses may be protective in normal cells, in cancer cells, they are often adapted to support survival, proliferation, and resistance to therapy [3]. Among these microenvironmental challenges, hypoxia, characterized by reduced oxygen availability in tissues, plays a particularly critical role. As tumours grow beyond the limits of their vascular supply, hypoxic regions inevitably arise, contributing to cancer cell adaptation and the selection of more aggressive phenotypes [4].

Hypoxia initiates a transcriptional program largely orchestrated by hypoxia-inducible factors (HIFs), especially HIF-1α [5]. Under normoxic conditions, HIF-1α is hydroxylated on specific proline residues, leading to its rapid degradation via the proteasome [6]. However, in hypoxic conditions, this degradation is blocked, allowing HIF-1α to dimerize with HIF-1β, translocate to the nucleus, and activate target genes involved in angiogenesis, glycolysis, cell survival, and invasion [7]. In addition to errors arising during DNA replication and repair, genome integrity is frequently threatened by genotoxic stress, including oxidative damage, ultraviolet light, and exposure to chemical agents [8, 9].

A widely used agent in this context is cobalt chloride (CoCl₂), a well-characterized hypoxia-mimicking compound that stabilizes HIF-1α by inhibiting its prolyl hydroxylation and preventing its proteasomal degradation under normoxic conditions [10]. Beyond HIF stabilization, CoCl₂ is also known to induce reactive oxygen species (ROS), leading to oxidative stress and DNA damage [11]. This dual action makes CoCl₂ a powerful experimental tool to study stress-induced responses relevant to cancer biology, especially in the pursuit of understanding how hypoxic stress can induce the expression of DNA mutator APOBEC3 enzymes, a potent source of mutagenesis and genome instability in cancer cells.

The human APOBEC3 (A3) family consists of seven cytidine deaminases, among which only APOBEC3A (A3A) and APOBEC3B (A3B) are capable of editing genomic DNA [12–16]. These enzymes deaminate cytosines to uracils in single-stranded DNA (ssDNA), a modification that is recognized and excised by uracil-DNA glycosylase (UNG). The removal of uracil generates abasic sites, which are processed by the base excision repair (BER) pathway. However, during DNA replication, this process can lead to the accumulation of double-strand DNA breaks (DSBs) and drive genomic instability [15, 16]. Notably, only A3A has been shown to directly cause DSBs and trigger a DNA damage response [15–17], implicating it as a potential driver of mutagenesis and carcinogenesis.

Mitochondria are best known as the cell’s powerhouse, generating ATP through oxidative phosphorylation. However, beyond energy metabolism, they play central roles in apoptosis, calcium signalling, and innate immunity. Their unique bacterial ancestry and possession of their own mitochondrial DNA (mtDNA) make them potent signallers of cellular distress. When mitochondrial integrity is compromised by toxins, hypoxia, or metabolic imbalance, these organelles become active participants in stress responses, releasing molecular alarms such as cytochrome c and mtDNA into the cytosol [18]. These signals can activate programmed cell death and inflammatory pathways, positioning mitochondria as both guardians of cell survival and arbiters of its demise.

The presence of cytosolic mtDNA serves as a potent danger-associated molecular pattern (DAMP), triggering a broad array of innate immune sensing pathways [19–23]. Once in the cytoplasm, mtDNA can be detected by several key receptors. The cGAS/STING/TBK1 pathway plays a central role, where cGAS binds cytosolic double-stranded DNA (dsDNA) and activates STING, leading to robust production of type I interferons (IFN) and pro-inflammatory cytokines [20, 24, 25]. Endosomal TLR9 also senses unmethylated CpG motifs abundant in mtDNA, initiating MyD88-dependent NF-κB signalling [26–28]. In parallel, AIM2 recognizes cytosolic dsDNA and forms an inflammasome complex, activating caspase-1 and promoting the maturation of IL-1β and IL-18 [29, 30]. Interestingly, mtDNA can also engage RNA-sensing pathways when transcribed into RNA species by RNA polymerase III (RNA pol III) [19, 31–33]. These transcripts could then be detected by RIG-I or MDA5, classical sensors of viral RNA, which further drive type I IFN responses through the MAVS signalling axis [19, 21, 33].

While IFN signalling is a well-characterized inducer of *A3A*, recent studies suggest that *A3A* can also be upregulated through IFN-independent mechanisms [34, 35]. Notably, cellular stress can trigger *A3A* expression even in the absence of detectable type I IFN activity, pointing to alternative regulatory pathways. Crucially, recent findings highlight the pivotal role of the NF-κB pathway, particularly its p65 subunit, in driving the transcriptional activation of *A3A* [35]. p65 is a key subunit of the NF-κB transcription factor complex, which regulates genes involved in inflammation, immune responses, cell survival, and stress signalling. Under stress conditions such as hypoxia or inflammation, p65 is activated and translocates into the nucleus, where it binds to κB consensus sites in the *A3A* promoter and enhances its transcription [35]. This process provides a mechanistic link between external stress signals and the endogenous mutagenic machinery of the cell.

Although *A3A* is traditionally considered an IFN-stimulated gene, our data show that hypoxia can induce *A3A* expression independently of type I IFN signalling. Cobalt chloride mimics hypoxia, causing mitochondrial stress, mtDNA release, and activation of the NF-κB pathway via p65, linking mitochondrial disturbance directly to *A3A* upregulation. This IFN-independent pathway highlights A3A as a key mediator of genotoxic stress. Overall, these findings demonstrate that hypoxia and related stress factors can repurpose an antiviral enzyme to promote genomic instability, potentially contributing to inflammation-driven diseases, metabolic disorders, and cancers.

## Materials and methods

### Reagents

RNA polymerase III inhibitor (ML-60218), digitonin (D141), RIPA Lysis Buffer 10X (20-188), Etoposide (341 205) were from Merck Millipore, cGAS inhibitor (G140) was from InvivoGen, TURBO DNase (2 U/μl) was from Invitrogen, Phenylmethanesulfonyl Fluoride (PMSF) (#8553) was from Cell Signalling Technology. HIF-1a (E1V6A; rabbit antibody mAb#48 085), Bak (D4E4; rabbit antibody mAb #12 105), Bax (D2E1, rabbit antibody mAb #5023), Bcl-2 (D17C4, Rabbit antibody mAb #3498), Cleaved Caspase-3 ((Asp175) (5A1E) rabbit antibody mAb #9664), MDA5 (D74E4; rabbit antibody mAb#5321), STING (D2P2F, rabbit antibody mAb#13 647), phospho-IRF-3 ((Ser396) (4D4G); rabbit antibody mAb#4947), IRF-3 (D6I4C; XP rabbit antibody mAb#11 904), RIG-I (D14G6; rabbit mAb#3743), MAVS (rabbit antibody #3993), TBK1/NAK (D1B4, rabbit antibody mAb#3504), phospho-TBK1/NAK ((Ser 172) (D52C2), XP rabbit antibody mAb#5483), IKKε (D20G4, rabbit antibody mAb#2905), phospho-IKKe ((Ser172) (D1B7), rabbit antibody mAb#8766), NFκB p65 (D14E12, XP rabbit antibody mAb#8242), IkBα (L35A5, mouse antibody (Amino-terminal Antigen) mAb#4814), MEK1/2 (D1A5, rabbit antibody mAb #8727), AIF (D39D2; XP rabbit antibody mAb #5318), STAT2 (D9J7L, rabbit antibody mAb #72 604), Phospho-STAT2 ((Tyr690) (D3P2P) rabbit antibody mAb #88 410), mCherry (E5D8F, rabbit antibody #43 590), β-actin horseradish peroxidase (HRP) conjugate (13E5, rabbit antibody mAb#5125), and Phospho-Histone H2A.X ((Ser139) (20E3) rabbit antibody mAb #9718) antibodies were from Cell Signalling Technology. Alexa Fluor 555 phalloidin (A34055) and MitoTracker Red CMXRos (M7512) were from Invitrogen. Alexa Fluor 647 Phalloidin (#8940S) was from Cell Signalling. Monoclonal Alexa Fluor 647 Mouse anti-H2AX (pS139) (#560 447) was from BD Biosciences. Monoclonal anti-mouse TFAM (18G102B2E11), anti-mouse Alexa Fluor 488 (#A32723), and anti-Rabbit Alexa Fluor 647 (#A21244) secondary antibody were from Thermo Fischer Scientific. The nucleus was stained using Hoechst dye (Invitrogen), and the samples were mounted in Fluoromount-G. THP-1 cells were labelled with MitoTracker Red and subsequently fixed with 4% paraformaldehyde (PFA). Following fixation, the cells were immunostained, and confocal z-stack images were acquired using a Zeiss LSM780 confocal microscope (ZEN acquisition software) equipped with a 63 × Plan-Apochromat 1.4 NA objective lens.

### Cell culture

THP-1 cells (ATCC TIB-202) were maintained in Roswell Park Memorial Institute medium (RPMI 1640, Gibco), supplemented with heat-inactivated fetal calf serum (10%), penicillin (50 U/mL), streptomycin (50 μg/ml), and β-mercaptoethanol (0.05 mM) and grown in 150-cm^2^ cell culture flasks. All experiments were performed with a low passage number of cells. QT6 cells (Japanese quail muscle fibroblast cell line, ATCC CRL 1708) were maintained in HAM’s F10 medium (Gibco), containing 1% chicken serum, 10% FCS, 5% tryptose phosphate, penicillin (50 U/ml), and streptomycin (50 μg/ml). Cells were cultured at 37.0°C under a humidified atmosphere containing 5% CO_2_.

### Construction of THP-1 DNase wild-type cell line

From the lab collection, a plasmid harboring the sequence of the cDNA of the wild-type DNase I gene, previously designed by GeneCust, was cloned into pcDNA3.1/V5 His-TOPO under the control of the CMV promoter, and the mCherry plasmid was cloned in pCR2.1 vector. Both were digested and ligated together. Ligated plasmid pcDNA 3.1 DNase (WT)-mCherry was used to insert the sequence of interest inside the lentiviral vector pTRIP harboring the promoter EF1α. HEK-293T cells were used to produce lentiviral vectors upon Fugene HD (Promega) transfection of DNase-mCherry vectors along with a p8.74 packaging plasmid and a VSV-G envelope-encoding plasmid. Then, THP-1 cells were transduced with lentiviral vectors purified from HEK-293T supernatants. mCherry-positive cells were sorted and amplified for establishing stable cell lines.

### SiRNA transfection

2.10^5^ THP-1 cells were plated in six-well tissue culture plate in 1ml antibiotic-free growth medium supplemented with fetal bovine serum (FBS). Cells were transfected according to standard protocol (Santa Cruz Biotechnology) with siRNA specific for RNA pol III_1_ (sc-76188, Santa Cruz Biotechnology) and RNA pol III_2_ (sc-36292, Santa Cruz Biotechnology) and control siRNA (sc-37007, Santa Cruz Biotechnology) or siRNA specific for APOBEC3A (HSS153372, HSS153373, HSS153374) or siRNA specific for HIF-1α (HSS104774, HSS104775, HSS179231) and Stealth RNAi Negative Control Duplexes (462 002) (from Thermo Fisher Scientific), using siRNA transfection reagent (sc-29528, Santa Cruz Biotechnology) or Lipofectamine™ RNAiMAX (Thermo Fisher Scientific). Twelve hours post-transfection with siRNA, cells were treated with 200 μM cobalt (II) chloride (CoCl_2_) (Sigma). At 20 h, cells were collected for further analysis.

### Total protein extraction and western blot analysis

Cells were treated with lysis buffer (0.5 M Tris-HCl, pH 7.4, 1.5 M NaCl, 2.5% deoxycholic acid, 10% NP-40, 10 mM EDTA, and EDTA-free Protease Inhibitor (Millipore)) followed by sonication and centrifugation at 20 000 x g for 20 min. Total cell lysates were loaded on NuPAGE^TM^ 4–12% Bis-Tris Gel (Invitrogen) and transferred onto a nitrocellulose membrane (Invitrogen) using iBlot 2 Dry Blotting System (Invitrogen) according to the manufacturer’s protocol. Membranes were blocked with phosphate buffer saline (PBS)-0.1% Tween, 5% Bovine Serum Albumin (BSA). After blocking, membranes were incubated with primary antibodies overnight at 4°C or directly incubated with HRP-conjugated antibody at room temperature for 2 h, washed 5 times with TBS, and incubated with the HRP-coupled secondary antibodies for 1 h at room temperature. SuperSignal™ West Pico chemiluminescent substrate (Thermo Fisher Scientific) was used to visualize proteins according to the manufacturer’s recommendation. Detection of β-actin served as a loading control.

### Subcellular fractionation

5.10^6^ cells were collected 20 h post-treatment, washed in PBS, and split. 10^6^ cells were kept for Western Blot and DNA extraction on whole cell extract (WCE), and 4.10^6^ cells were lysed in 0.5 ml digitonin buffer (150 mM NaCl, 50 mM Hepes (pH 7.4), 0.03 mM, protease and phosphatase inhibitors cocktail) for 10 min at 4˚C. After centrifugation (2000 g, 10 min, 4˚C), the supernatant was centrifuged three times (20 000 g, 20 min, 4˚C). The last supernatant constitutes the cytosolic fraction and was split into two: one half for DNA extraction, the other for western blotting. The initial pellet was resuspended in 300 μL NP-40 buffer (150 mM NaCl, 50 mM HEPES pH7.4, 1% NP-40, protease and phosphatase inhibitors cocktail), for 30 min at 4˚C. After centrifugation (7000 g, 10 min, 4˚C), the pellet and supernatant were split in half for western blotting and DNA extraction; the pellet constitutes the nuclear fraction, and the supernatant the crude mitochondrial lysate.

### Flow cytometric analysis of DSBs and apoptosis

The analysis of DSBs is in THP-1 or QT6 cells when treated with CoCl_2_ and collected 20 h later. For double-strand DNA break analysis, cells were washed with PBS, fixed in 2% ice-cold paraformaldehyde (Electron Microscopy Sciences) for 10 min, and permeabilized in 90% ice-cold methanol (Sigma) for 30 min. After PBS washing, cells were incubated with 1:100 diluted Alexa Fluor 647 mouse anti-γH2A.X (pS139) antibody (BD Biosciences) in PBS-5% BSA for 1 h. For apoptosis analysis, THP-1 cells were collected and washed with PBS, stained with 7-AAD viability staining solution (1/40; eBioscience), then washed with annexin V binding buffer (eBioscience), and stained with anti-annexin V eFluor 450 (1/20, eBioscience). Treatments with 25 or 100 μM etoposide in dimethyl sulfoxide were used as positive control. All stained samples were acquired on a MACSQuant analyzer (Miltenyi Biotech), and data were analysed with FlowJo software (Tree Star, Inc.; version 8.7.1).

### Flow cytometric analysis of the release of cytochrome *c*

To evaluate the release of mitochondrial Cytochrome c in THP1 cells treated with CoCl_2_, cells were harvested at 20 h post-treatment. Cells were investigated for cytochrome c release using the FlowCellect Cytochrome c Kit from Millipore, following the manufacturer’s instructions. Cells were analysed using Cell Quest Pro (BD Biosciences) and FlowJo software (Tree Star, Inc., version 8.7.1). For each sample, 10 000 cells were counted.

### RNA extraction and quantitative reverse-transcription PCR (Taqman or Sybr Green)

THP-1 cells treated with drugs were collected, and total RNA was extracted using the RNeasy Plus Mini Kit (Qiagen) following the manufacturer’s instructions. RNA was eluted in 40 μl of RNase-free water. Subsequently, 1 μg of total RNA was used to synthesize cDNA using the QuantiTect Reverse Transcription Kit (Qiagen). To eliminate genomic DNA, gDNA wipeout buffer was added to the template RNA, then the mix was incubated for 5min at 42°C. The synthesis reactions of cDNA were performed by mixing 1 μl Quantiscript Reverse Transcriptase, 4 μl Quantiscript RT Buffer, 1 μl RT Primer Mix, and 14 μl of the entire genomic DNA elimination reaction, which were incubated at 42°C for 30 min. Expression levels of *A3A, A3B, A3C, A3DE, A3F, A3G, A3H, RPL13A, IFNα* and *IFNβ* were quantified by qPCR using an QuantStudio3 thermocycler (Thermo Fisher Scientific). The reactions were carried out in a 96-well plate, and for each well, the reaction consisted of 12.5 μl of TaqMan Fast Advanced Master Mix (Applied Biosystem), 2 μl of primers (5 μM each, Sigma), 0.33 μl of UPL probe (Roche), 5.17 μl of water, and 5 μl of complementary DNA. The qPCRs were performed as follows: for A3A through A3H, the conditions were: 95°C 10 min followed by 45 cycles (95°C 15 s, 58°C 15 s, 68°C 20 s), for IFNα and IFNβ, the conditions were: 95°C 10 min followed by 45 cycles (95°C 10 s, 58°C 10 s, 68°C 30 s). The data obtained from different samples were normalized with respect to RPL13A expression levels. MtDNA (*MTF3212* and *MT-COI*) was quantified using quantitative PCR based on SYBR green (Applied Biosystems). The reactions were carried out in a 96-well plate, and for each well, the reaction consisted of 12.5 μl of Power SYBR Green PCR Master Mix (Applied Biosystem), 2 μl of primers (5 μM each, Sigma), 5.5 μl of water, and 5 μl of complementary DNA. Conditions were 2 min at 50°C and 10 min at 95°C, followed by 40 cycles (95°C 15, 55°C 15 s, 68°C 1 min), followed by a melting curve step. Data were normalized to the expression levels of the nuclear reference gene *β2M. APOBEC3A-H* primers have already described [36], Supplementary Table S2 lists all other primers and probe sequences.

### RNA-sequencing data processing and statistical analysis

Raw sequencing data were assessed using FastQC (v0.12.1) [37] and MultiQC (v1.12) [38]. Reads were trimmed to improve low-quality bases and adapter sequences using Trimmomatic (v0.39) [39] using the parameters: ILLUMINACLIP: TruSeq3-PE.fa:2:30:10; LEADING:3; TRAILING:3; SLIDINGWINDOW:4:20; MINLEN:36. Trimmed reads were aligned to the human reference genome (GENCODE_GRCh38.p14) using STAR alignment tool (v2.7.11b) [40]. Alignment quality metrics were summarized using MultiQC (v1.12) [38], and gene counts were extracted using the featureCounts function from the Subread package (v2.0.0) [41]. Differential expression analysis was performed in R (v4.4.3) within the RStudio environment using the DESEQ2 package (v1.46.0) [42]. Differentially Expressed Genes. (DEGs) were identified with a threshold of adjusted *P*-value < 0.0.5 and log fold change > 1.5. DEGS were subjected to Gene Ontology (GO) enrichment analysis for Biological Processes (BP) using the clusterProfiler package (v4.14.6) [43] and to Gene Set Enrichment Analysis (GSEA) using the fgsea package (v1.32.4) [44]. Data visualizations were performed using base R, ggplot2 (v4.4.0) [45] and pheatmap (v1.0.13) [46].

## Results

### Cobalt chloride-induced hypoxia promotes *A3A* expression in THP-1 cells.

**Figure 1.**
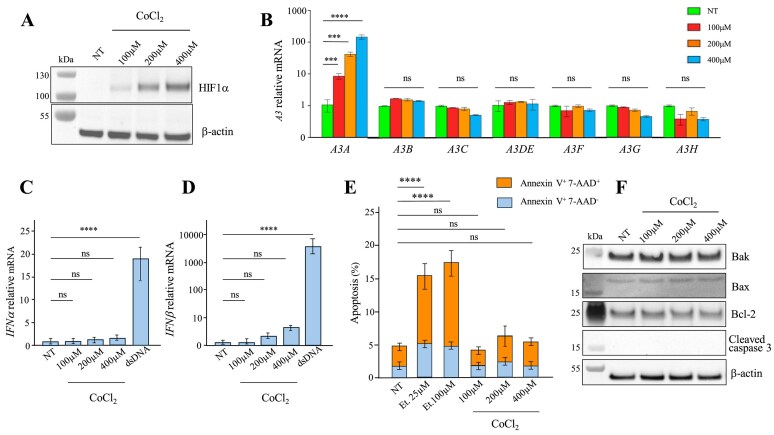
Effects of CoCl₂ on *HIF-1α, A3* expression, and apoptosis markers in THP-1 cells. (**A**) Detection of HIF-1α protein by western blot in THP-1 cells treated with 100, 200, and 400 μM CoCl₂, compared with untreated controls (NT). β-actin was used as a loading control. Each blot was repeated twice. (**B**) Relative expression of *A3* in THP-1 cells treated with 100, 200, and 400 μM CoCl₂ for 20 h normalized to *RPL13A*. (**C-D**) Relative expression of *IFNα and IFNβ* in THP-1 cells treated with 100, 200, and 400 μM CoCl₂ and double-stranded DNA used as a positive control for 20 h. Mean values ± SEM were calculated from three independent experiments performed in duplicate (*n* = 6). (**E**) Flow cytometry analysis using annexin V and 7-AAD staining after 20 h of treatment with 100, 200, and 400 μM CoCl₂. Mean values ± SEM were calculated from three independent experiments (*n* = 3). (**F**) Detection of Bak, Bax, Bcl-2, and cleaved caspase-3 proteins by western blot in THP-1 cells treated with 100, 200, and 400 μM. CoCl₂, compared with untreated controls. β-actin was used as a loading control. Each blot was repeated twice. For B–E, data obtained were subjected to two-way ANOVA, followed by an *ad hoc* test, * *P* < 0.05, **P < 0.01, *** *P* < 0.001, and **** *P* < 0.0001, ns: not statistically significant.

Hypoxia triggers a variety of cellular stress responses, but its impact on *A3* expression still remains unclear. Cobalt chloride, a widely used chemical hypoxia-mimicking, stabilizes HIF-1α by inhibiting prolyl hydroxylase enzymes [10, 47]. THP-1 cells, a human monocytic leukaemia cell line, were selected due to their physiological relevance in hypoxic conditions, as monocytes and macrophages are frequently exposed to low-oxygen environments in both healthy and pathological tissues [48]. To investigate the relationship between hypoxia and *A3* expression, THP-1 cells were treated for 20 h with increasing concentrations of cobalt chloride at 100, 200, and 400 μM and compared to untreated cells (NT). As shown in Fig. 1A, HIF-1α protein levels increased in a dose-dependent manner, confirming the induction of hypoxic conditions.

To assess whether *A3s* were upregulated under hypoxic stress, THP-1 cells were incubated with the same increasing cobalt chloride concentrations, and *A3* expressions were analysed. Following RNA extraction and cDNA synthesis, transcriptional profiling of the *A3* gene family, normalized to the *RPL13* housekeeping gene, revealed a selective and pronounced induction of *A3A*, which increased proportionally with cobalt chloride concentration by approximately ∼1–2 log when compared to untreated conditions (Fig. 1B). In contrast, the expression of *A3B, A3C, A3DE, A3F, A3G* and *A3H* remained stable. These results indicate that cobalt chloride activates signalling pathways, likely related to hypoxic or oxidative stress, that drive dose-dependent transcription of *A3A*. Importantly, treatment with 100, 200, or 400 μM CoCl₂ did not induce the expression of *IFNα* or *IFNβ* (Fig. 1C and D), nor did it activate the canonical JAK-STAT pathway, which depends on STAT phosphorylation and dimerization (Supplementary Fig. S1A). As a positive control, THP-1 cells were transfected with 500ng of dsDNA, a well-characterized inducer of type I IFNs. After 20 h, RT-qPCR analysis revealed an induction of *IFNα* and *IFNβ* expressions by ∼18-fold and ∼6000-fold, respectively (Fig. 1C and D). These results confirm that THP-1 cells mounted a robust type I IFN response under these conditions, validating the integrity of the assay.

We next examined whether cobalt chloride leads to apoptosis. THP-1 cells were treated with the same increasing concentrations of cobalt chloride for 20 h, and apoptosis was assessed by flow cytometry using annexin V and 7-AAD staining (Fig. 1E). The assay distinguishes between early apoptotic cells (Annexin V^+^/7-AAD^-^) and late apoptotic or necrotic cells (Annexin V^+^/7-AAD^+^). As shown in Fig. 1E, none of the cobalt chloride concentrations induced significant apoptosis when compared with untreated cells. Inversely, etoposide-treated positive controls triggered ∼3-fold increases in apoptosis. Supporting this observation, western blot analysis of apoptotic regulators, including Bcl-2, Bax, Bak, and cleaved caspase-3, showed no detectable differences in protein levels between treated and untreated cells (Fig. 1F).

Taken together, these findings demonstrate that hypoxic stress alone potently induces *A3A* transcription in a dose-dependent manner. This induction is uncoupled from IFN signalling and occurs without apoptosis, suggesting that hypoxia engages a distinct regulatory pathway capable of driving *A3A* expression independently of canonical IFN responses.

### Chromosomal DNA damage induced by cobalt chloride is potentially driven by endogenous A3A

**Figure 2.**
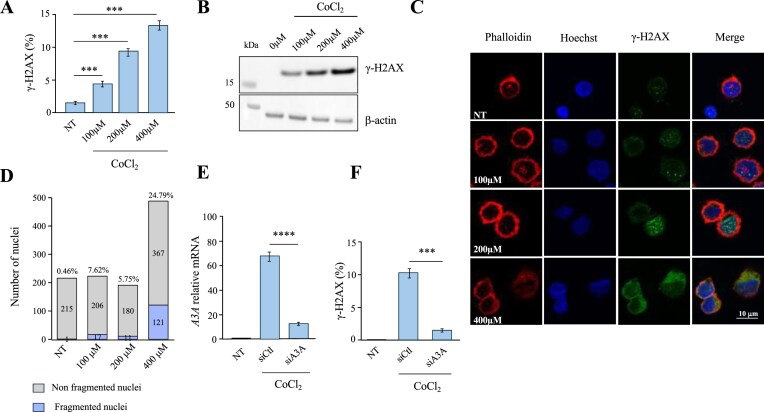
CoCl₂-induced DNA damage and A3A regulation in THP-1 cells. (**A**) Flow cytometry analysis of γH2AX-positive THP-1 cells treated with 100, 200, and 400 μM CoCl₂ for 20 h and compared with untreated controls. (**B**) Detection of γH2AX protein by western blot in THP-1 cells treated with 100, 200, and 400 μM CoCl₂, compared with untreated controls. β-actin was used as a loading control. Each blot was repeated twice. (**C**) Immunofluorescence analysis of THP-1 cells treated or untreated with CoCl_2_. Actin filaments were stained with phalloidin, nuclei with DAPI, and DNA double-strand breaks with anti-γH2AX. (**D**) Analysis of the ratio of fragmented to non-fragmented nuclei in THP-1 cells treated with 100, 200, and 400 μM, compared with untreated controls. The histograms display both the fragmented and non-fragmented number of nuclei analysed and the corresponding percentage of fragmented nuclei. (**E**) Relative expression of *A3A* in THP-1 cells treated with 200 μM CoCl₂ and transfected with sicontrolRNA or siA3A, compared with untreated controls. Error bars represent the standard deviation from three independent experiments. (**F**) Flow cytometry analysis of γH2AX-positive THP-1 cells treated with 200 μM CoCl₂ and transfected with sicontrol RNA or si*A3A* RNA, compared with untreated controls. Data obtained in A, E, and F were subjected to two-way ANOVA, followed by an *ad hoc* test, * *P* < 0.05, **P < 0.01, *** *P* < 0.001, and **** *P* < 0.0001, ns: not statistically significant.

Given that the primary function of A3A is to deaminate DNA, a process that can lead to DSBs [15, 49], we investigated whether cobalt chloride treatment could induce DSBs, potentially through the upregulation of *A3A*. To assess the impact of *A3A* induction, THP-1 cells were treated with increasing concentrations of CoCl₂, at 100, 200, and 400 µM. Following treatment, we observed that γ-H2AX levels, a marker of DSBs, increased with CoCl₂ concentrations, reaching ∼5%, ∼10%, and ∼14%, respectively (Fig. 2A). These findings were further confirmed by western blot analysis showing a similar γ-H2AX increase (Fig. 2B). Immunofluorescence further validated these results using phalloidin to label the actin cytoskeleton, anti-γ-H2AX antibodies to detect DNA damage, and Hoechst dye to stain nuclei (Fig. 2C). Importantly, the occurrence of DSBs was associated with a significant increase in genomic instability, in a dose-dependent manner of CoCl₂ concentration (Fig. 2C). Analysis of nuclear fragmentation across increasing CoCl₂ concentrations also showed a significant occurrence of nuclear disorganization, with ∼24.8% of cells displaying a destructured nucleus at 400 μM CoCl₂ (Fig. 2D).

To further validate this hypothesis, *A3A* expression using specific siRNAs was silenced. Hence, THP-1 cells were transfected with either *A3A*-targeting siRNAs or a non-targeting control siRNA. At 12 h post-transfection, cells were exposed to 200 µM CoCl₂ for 20 h, a concentration commonly used to mimic hypoxic conditions *in vitro* and at which *A3A* expression is significantly upregulated (Fig. 1B). The efficacy of *A3A* knockdown was confirmed by RT-qPCR, which revealed ∼4-fold reduction in *A3A* mRNA levels in cells transfected with *A3A* siRNA compared to control siRNA (Fig. 2E). This reduction was associated with a ∼80% decrease in DSBs (Fig. 2F), indicating that A3A plays a key role in mediating the observed DNA damage.

To determine whether CoCl_2_-induced DNA damage was specifically attributable to the cytidine deaminase activity of A3A, we performed parallel experiments in QT6 cells, quail fibroblasts that naturally lack A3 proteins [16]. Notably, QT6 cells exposed to 200 μM cobalt chloride showed no detectable γ-H2AX signal by Flow cytometry analysis, whereas etoposide-treated cells exhibited ∼60-fold increase (Supplementary Fig. S1B), indicating that DSBs observed under these conditions are dependent on A3A enzymatic activity. Taken together, these findings demonstrate that hypoxia-induced A*3A* expression is directly responsible for the generation of DSBs in THP-1 cells treated with cobalt chloride.

### Hypoxia-driven transcriptional reprogramming highlights mitochondrial adaptation and stress response pathways

**Figure 3.**
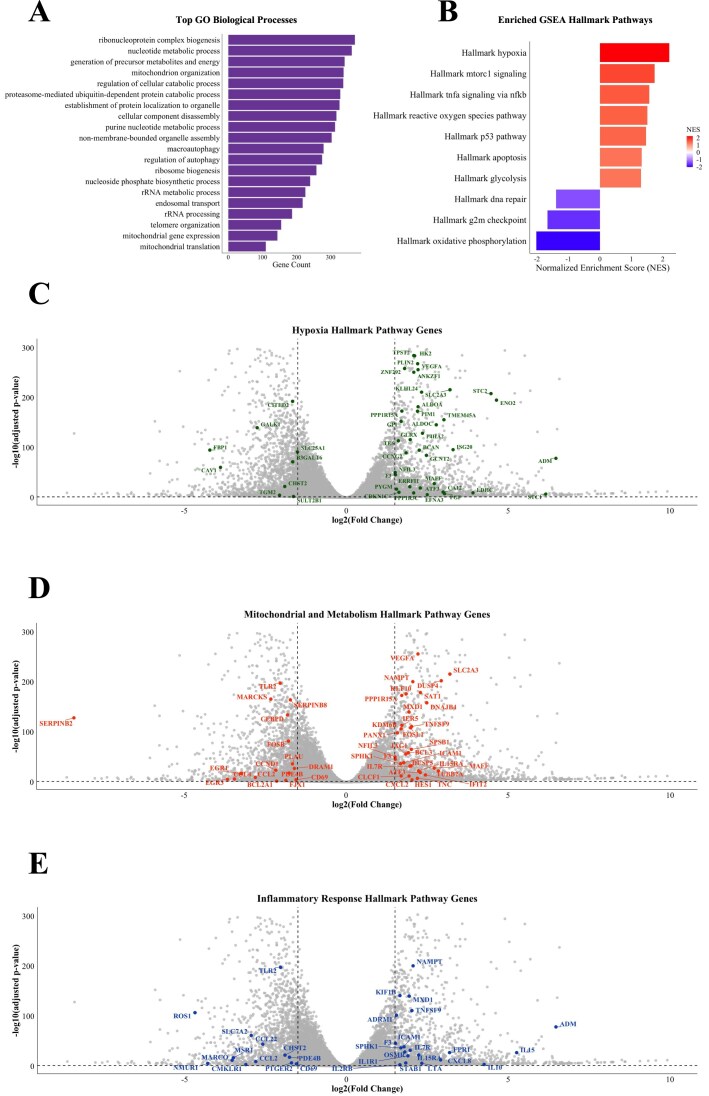
CoCl₂-induced differential gene expression and mitochondrial pathway regulation. (**A**) GO Biological Processes enriched among differentially expressed genes (DEGs), identified using a threshold of adjusted *P*-value < 0.05 and log_2_ fold change (log₂FC) ≥ 1.5. The top enriched processes are listed on the *y*-axis, with the number of genes associated with each biological process shown on the *x*-axis. (**B**) Gene Set Enrichment Analysis (GSEA) Hallmark pathways enriched among the DEGs. The Normalized Enrichment Score (NES), displayed on the *x*-axis, reflects which pathway is represented. Positive NES values indicate pathways enriched in upregulated genes, while negative NES values indicate enrichment in downregulated genes. (**C-E**) Volcano plots displaying genes belonging to Hallmark pathways from GSEA. Each plot shows the log_2_FC (*x*-axis) versus –log_10_ (*P*-value) (*y*-axis). Vertical dashed lines indicate the log₂FC thresholds (log_2_FC ≥ 1.5), and the horizontal line marks the significance threshold corresponding to *P *= 0.05. Non-significant genes in all panels are shown in grey. (**C**) Hypoxia Hallmark pathway genes. (**D**) Mitochondrial and metabolic pathways, including Oxidative Phosphorylation, Glycolysis, and Reactive Oxygen Species Pathway. (**E**) Inflammatory Response Hallmark pathway genes.

To identify hypoxia-induced secreted factors, RNA-sequencing (RNAseq) was performed on THP-1 cells treated with 200 µM CoCl₂, and gene expression profiles were compared to those of untreated controls. All samples were normalized using the median-to-ratios approach implemented in DESEQ2 (Supplementary Fig. S2A). Principal Component Analysis (PCA) was performed to examine the overall variance in gene expression profiles between samples. The first principal component (PC1) captures the largest source of variance (99%), separating the control and CoCl_2_-treated samples, indicating a significant change in the transcriptomic profile between treated and untreated samples (Supplementary Fig. S2B). This difference was further emphasized by the top variable genes between samples (Supplementary Fig. S2C). Gene Ontology (GO) enrichment analysis performed on all significant genes (*P *< 0.05) with a log_2_ fold change (log₂FC) ≥ 1.5, using the R package Cluster Profiler [50], reinforced the central role of mitochondrial adaptation by revealing enriched biological processes such as mitochondrial translation, gene expression, mitochondrial organization, and autophagy (Fig. 3A).

To uncover potential functional associations between genes and cellular processes, a Gene Set Enrichment Analysis (GSEA) was performed using the fgsea R package [44] to identify hallmark pathways significantly enriched among the upregulated and downregulated genes between conditions (Fig. 3B, Supplementary Table S1). Hypoxia emerged as a primary driver of the transcriptional response, with Differentially Expressed Genes (DEGs) strongly enriched in hypoxia-related programs, reactive oxygen species (ROS) signalling, glycolysis, apoptosis, and DNA repair pathways. Notably, many genes within mitochondrial pathways, including those involved in oxidative phosphorylation and energy metabolism, were downregulated (Fig. 3 and Supplementary Fig. S2), reflecting a profound alteration of the mitochondrial network. These transcriptional changes indicate that cells actively reprogram their metabolism and energy production to cope with chemical hypoxia.

DEGs were identified using the R package DESEQ2 [42], with a total of 2287 and 1983 human genes significantly upregulated and downregulated, respectively (adjusted *P*-value < 0.05, |log₂ fold change| > 1.5) (Fig. 3 and Supplementary Fig. S2). Volcano plots generated using the R package ggplot2 [45] illustrate the global distribution of DEGs associated with hypoxia, mitochondrial and metabolic pathways, and inflammatory responses (Fig. 3C–E, Supplementary Table S1). Each point represents an individual gene, plotted by fold change (*x*-axis) and statistical significance (*y*-axis), providing a comprehensive overview of the transcriptional landscape under chemical hypoxia. Genes belonging to key functional pathways were highlighted in distinct colours: hypoxia-responsive genes (green, Fig. 3C), mitochondrial and metabolism genes (red, Fig. 3D), and inflammatory response genes (blue, Fig. 3E). This visualization emphasizes both the magnitude and pathway-specific patterns of transcriptional changes, underscoring coordinated regulation of hypoxia signalling, mitochondrial function, immune activation, and DNA maintenance in response to CoCl₂. Downregulation of specific mitochondrial genes suggests a strategic adjustment of metabolic activity to withstand hypoxic stress (Fig. 3).

Collectively, these results demonstrate that CoCl₂-treated THP-1 cells undergo a coordinated transcriptional reprogramming focused on mitochondrial adaptation, stress-response activation, and DNA maintenance, highlighting the critical interplay between energy metabolism and adaptive signalling under hypoxia.

### Cobalt chloride-induced hypoxia disrupts mitochondrial network organization and promotes cytosolic mtDNA release

**Figure 4.**
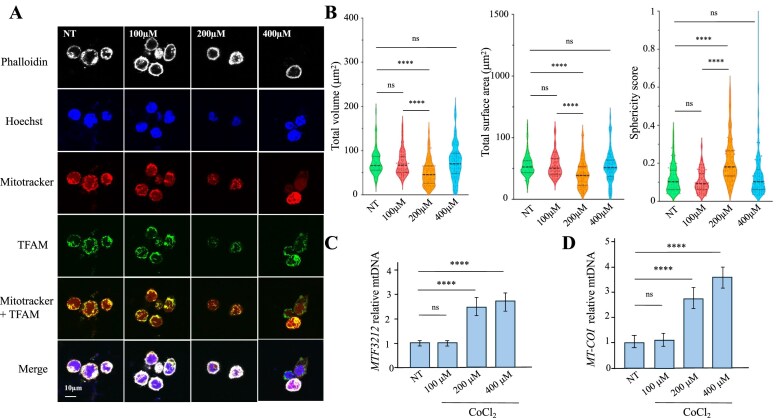
Mitochondrial Remodelling and Bioenergetic Alterations in CoCl₂-Treated THP-1 cells. (**A**) Immunofluorescence analysis of THP-1 cells treated with 100, 200, or 400 μM CoCl₂ for 20 h, compared with untreated controls, using confocal microscopy. Actin filaments were stained with phalloidin, nuclei with Hoechst, mitochondria with MitoTracker, and mtDNA with an anti-TFAM monoclonal antibody. (**B**) Quantification of total mitochondrial volume and surface area normalized to nuclear volume, based on confocal images. Box plots were generated for NT cells, 100 μM CoCl_2_, 200 μM CoCl_2_, and 400 μM CoCl_2_. Mitochondrial sphericity was also evaluated, with values approaching 1 indicating near-spherical morphology and values closer to 0 indicating deviation from sphericity. (**C-D**) Fractionation of THP-1 cells at 20 h post-treatment followed by quantification of mtDNA using *MTF3212* (*MT-LT1*, mitochondrially encoded tRNA-Leu) and *MT-COI* (cytochrome c oxidase subunit I) in the cytosolic fraction, normalized to the nuclear gene *β2M* in the total fraction. Mean values ± SEM were calculated from three independent experiments performed in duplicate (*n* = 6). Data obtained in B, C, and D were subjected to two-way ANOVA, followed by an *ad hoc* test, * *P* < 0.05, **P < 0.01, *** *P* < 0.001, and **** *P* < 0.0001, ns: not statistically significant.

Mitochondria are central to maintaining cellular homeostasis and coordinating stress responses. Cobalt chloride may exert its effects by altering mitochondrial dynamics and promoting membrane permeabilization. To investigate this, THP-1 cells were exposed to 100, 200, and 400 µM CoCl₂ and compared with untreated cells. As observed in Fig. 4A, mitochondria and cellular structures were stained using mitotracker (dye for mitochondria labelling), anti-TFAM (Mitochondrial transcription factor A) and phalloidin labelling probe, while nuclei were visualized with Hoechst dye. Compared to untreated cells, CoCl₂ treatment exhibits progressive remodelling of the mitochondrial network, as evidenced by changes in TFAM localization, which appeared more diffuse and less concentrated in the perinuclear region (Fig. 4A and Supplementary Fig. S3).

To statistically validate these observations, confocal microscopy images were treated using the MitoAnalyser plugin for Fiji, and quantitative parameters related to total mitochondrial volume and surface area were analysed. As shown in Fig. 4B, mitochondrial morphology was quantified in CoCl₂-treated cells and compared with randomly selected untreated controls. Notably, cells exposed to 200 μM CoCl₂ exhibited an approximately ∼1.5-fold and ∼1.3-fold reduction in mitochondrial volume and surface area, respectively, compared with untreated cells or cells treated with 100 and 400 μM. The reduction in mitochondrial volume and surface area was accompanied by an increase in mitochondrial sphericity, consistent with enhanced mitochondrial network fragmentation under CoCl₂ treatment (Fig. 4B). Interestingly, exposure to 400 μM CoCl₂ causes marked disruption and degradation of the mitochondrial network (Fig. 4A), while overall mitochondrial volume and surface area remained nearly unchanged relative to untreated cells (Fig. 4B). This apparent paradox may reflect the strong toxicity of CoCl₂ at this concentration, which depletes the majority of mitochondria while sparing a resistant subset. In line with this protective adaptation, exposure to 200 µM CoCl₂ resulted in the downregulation of several metal ion transporters and exporters, including *SLC39A8, SLC39A14*, and *SLC40A1* genes, reduced by ∼2.1-fold, ∼4.8-fold, and ∼7.7-fold, respectively (Supplementary Fig. S1C). Such suppression likely represents an active strategy by the cell to limit cobalt entry, thereby mitigating metal-induced toxicity and preserving mitochondrial and cellular viability.

To further explore the link between hypoxia and inflammatory signalling, we examined the cytosolic release of mtDNA. Under hypoxic stress, mitochondrial membrane integrity can be compromised, facilitating the leakage of mtDNA into the cytosol, a key trigger of innate immune pathways [51]. To model this, THP-1 cells were exposed for 20 h to increasing concentrations of CoCl₂, 100, 200, and 400 µM. To quantify released mtDNA, cytosolic fractions were isolated via digitonin-based subcellular fractionation and analysed by immunoblotting (Supplementary Fig. S1E). The absence of the mitochondrial marker AIF and the presence of the cytosolic marker MEK1/2, together with β-actin as a control, confirmed the purity of the cytosolic fraction (Supplementary Fig. S1E). Quantification of mtDNA by qPCR targeting mitochondrial genes *MTF3212* (*MT-LT1*, mitochondrially encoded tRNA-Leu) and *MT-COI* (mitochondrially encoded cytochrome c oxidase I), normalized to nuclear *β2M* (Beta-2 microglobulin), revealed a dose-dependent increase in cytosolic mtDNA levels of ∼2.5-fold and ∼2.5–3.5-fold in cells treated with 200 and 400 μM CoCl₂, respectively, compared to controls (Fig. 4C and D). In parallel with the release of mtDNA, quantification of cytochrome c release revealed a clear dose-dependent response with ∼4-fold, ∼9-fold, and ∼23-fold increases, respectively (Supplementary Fig. S1D).

**Figure 5.**
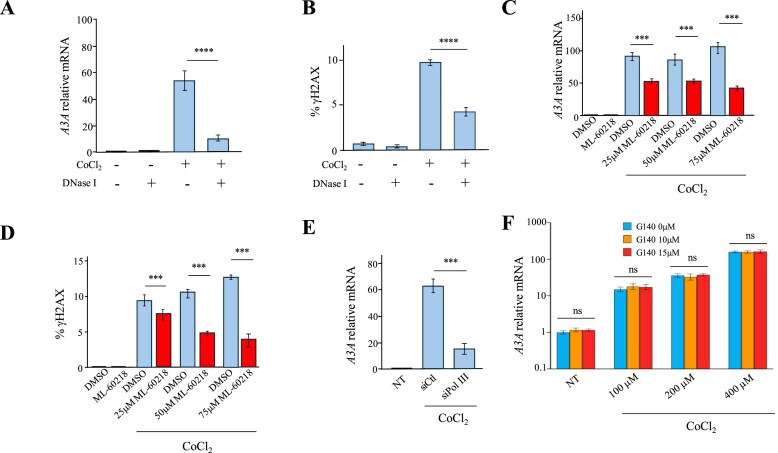
Mitochondrial DNA dynamics and A3A response in CoCl₂-treated THP-1 cells. (**A-B**) *A3A* expression by RT-qPCR and quantification of DNA double-strand breaks by flow cytometry at 20 h after treatment with 200 μM CoCl₂ in THP-1 cells expressing DNase I or not. Data were normalized to the *RPL13A* housekeeping gene. Mean values ± SEM were calculated from three independent experiments performed in duplicate (*n* = 6). (**C-D**) *A3A* relative expression (normalized to *RPL13A*) and quantification of DNA double-strand breaks by flow cytometry in THP-1 cells treated with 200 μM CoCl₂, with or without concomitant treatment with ML-60218 (RNA pol III inhibitor) at 25, 50, or 75 μM. (**E**) *A3A* relative expression in 200 μM CoCl₂-treated THP-1 cells transfected with control siRNA or siPolIII RNA, compared with untreated cells. Error bars represent the standard deviation from three independent experiments. (**F**) *A3A* relative expression (normalized to *RPL13A*) in THP-1 cells treated with 100, 200, 400 μM CoCl₂ in the presence of G140 (cGAS inhibitor) at 0, 10, or 15 μM. Data obtained in A-F were subjected to two-way ANOVA, followed by an *ad hoc* test, * *P* < 0.05, **P < 0.01, *** *P* < 0.001, and **** *P* < 0.0001, ns: not statistically significant.

To determine whether cytosolic mtDNA was the primary driver of *A3A* upregulation under hypoxic stress, a stable THP-1 cell line expressing DNase I-mCherry was generated by lentiviral transduction, followed by cell sorting based on mCherry fluorescence and validated by western blot (Supplementary Fig. S1F). If mtDNA contributes to *A3A* induction, it would be expected to be diminished in cell lines expressing DNase I. DNase I-expressing THP-1 cells and wild-type controls were treated with 200 μM CoCl₂, and *A3A* expression and DSBs were analysed at 20 h post-treatment. The DNase I-expressing cells exhibited a ∼6-fold and ∼2-fold reduction in *A3A* expression (Fig. 5A) and DSB, respectively (Fig. 5B), indicating that cytosolic mtDNA plays a significant role in *A3A* activation in response to hypoxic stress.

Overall, CoCl₂-induced hypoxia disrupts mitochondrial structure, triggering a dose-dependent release of mtDNA into the cytosol. This release alters the shape and volume of the mitochondrial network, conducting to reduced and clustered mitochondria around the nucleus. This process may represent a key mechanism through which mitochondrial stress initiates innate immune activation.

### Hypoxia-induced mitochondrial stress activates the A3A via RNA polymerase III/RIG-I/NF-κb signalling pathway

Consistent with previous observations, exposure of THP-1 to 100, 200, and 400 µM CoCl₂ exhibited a marked increase in *A3A* expression (Fig. 1B), occurring independently of type I IFN signalling (Fig. 1C and D). Since *A3A* is classically upregulated by type I IFNs [33, 52, 53], this unexpected response raised the possibility of alternative stress- or DNA-sensing pathways. Given that cobalt chloride acts as a hypoxia-mimicking agent, we tested the role of HIF1α in *A3A* induction. Treatment of cells with 200 µM CoCl₂ failed to enhance *HIF-1α* expression but stabilized the protein through inhibition of its proteasomal degradation, suggesting that *A3A* upregulation occurs independently of HIF-1α and reflects post-translational regulatory mechanisms (Supplementary Fig. S1G). In agreement, siHIF1α knockdown had no effect compared with control siRNA, confirming that *A3A* upregulation occurs independently of HIF-1α (Supplementary Fig. S1H).

Emerging evidence highlights mitochondrial dysfunction and the release of mtDNA into the cytosol as potent triggers of innate immune activation [19–23, 33]. While the canonical cGAS/STING/TBK-1 signalling cascade is a well-characterized DNA-sensing mechanism, mounting studies have also implicated an alternative RNA pol III/RIG-I pathway [19, 21, 31–33]. In this context, cytosolic dsDNA can be transcribed by RNA Pol III into immunostimulatory RNA species, which in turn activates should be replaced by "RIG-I receptors and triggers downstream immune signalling. Since STING sits at the crossroads of these pathways, we explored whether similar mechanisms might underlie *A3A* induction under CoCl₂-induced hypoxic stress.

To distinguish between the two DNA-sensing pathways, THP-1 cells were treated for 20 h with 200 µM CoCl_2_ in combination with ML-60218, a selective RNA Pol III inhibitor at 25, 50, and 75 µM [54]. DMSO-treated cells were used as controls. RT-qPCR analysis revealed a dose-dependent decrease in *A3A* expression, approximately ∼1.8-fold, ∼1.6-fold, and ∼2.4-fold reductions with increasing concentrations of ML-60218 (Fig. 5C). Consistent with this, DNA DSBs, a downstream consequence of A3A activity, were also reduced by ∼1.3-fold, ∼2.2-fold, and ∼2.7-fold in the presence of 25, 50, and 75 µM ML-60218, respectively (Fig. 5D). To further validate the involvement of RNA Pol III, siRNA-mediated knockdown of RNA Pol III subunits was performed (a combination of two siRNAs against RNA Pol III). Knockdown led to significant reductions in *A3A* expression ∼2.8-fold compared with control siRNA-treated cells, supporting RNA Pol III as a critical regulator of *A3A* expression under hypoxic conditions (Fig. 5E).

To investigate the contribution of cGAS, THP-1 cells were exposed to 100, 200, and 400 μM CoCl₂ in the presence of 10 or 15 μM G140, a cGAS pathway inhibitor [55]. As shown in Fig. 5F, inhibition of cGAS had no effect on *A3A* expression, indicating that this pathway operates independently of cGAS signalling.

**Figure 6.**
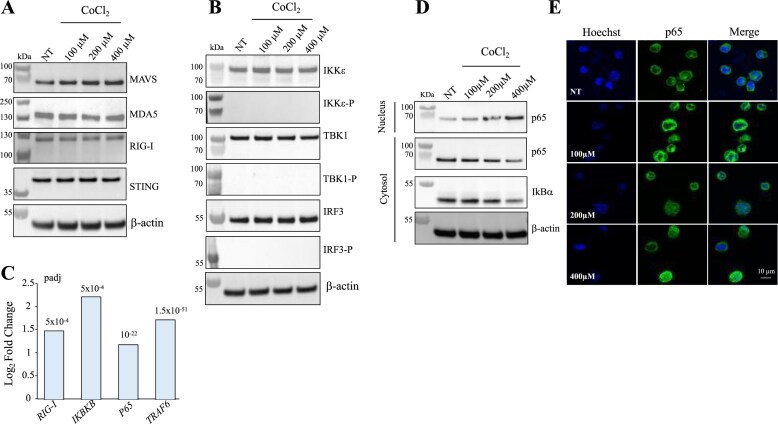
A3A regulation and innate immune signalling in CoCl₂-treated THP-1 cells. (**A**) Western blot analysis of MAVS, MDA5, RIG-I, and STING at 20 h post-treatment of THP-1 cells with 100, 200, or 400 μM CoCl₂, compared with untreated cells. β-actin was used as a loading control. (**B**) Western blot analysis of IKKε, phospho-IKKε, TBK1, phospho-TBK1, IRF3, and phospho-IRF3 at 20 h post-treatment of THP-1 cells with 100, 200, or 400 μM CoCl₂, compared with untreated cells. β-actin was used as a loading control. (**C**) RNAseq data were extracted from Supplementary Table S1 to show the Log_2_ fold change of *RIG-I, IKBKB, p65*, and *TRAF6* expression from THP-1 treated with 200 μM CoCl_2,_ following DGE. (**D**) Western blot analysis of nuclear and cytosolic fractions of THP-1 cells treated with 100, 200, or 400 μM CoCl₂, compared with untreated cells, probing for p65 and IκBα. β-actin was used as a loading control. (**E**) Immunofluorescence analysis of THP-1 cells treated with 100, 200, or 400 μM CoCl₂ for 20 h, compared with untreated controls. Nuclei were stained with Hoechst, and p65 was visualized with an anti-p65 monoclonal antibody using confocal microscopy.

Western blot analysis was performed on THP-1 cells treated with increasing concentrations of CoCl₂ to assess the potential activation of cytosolic nucleic acid-sensing pathways. The expression of MAVS, MDA5, RIG-I, and STING was clearly detectable in both untreated and CoCl₂-treated cells; however, their levels remained stable across all treatment conditions, indicating that hypoxic stress did not significantly modulate these key sensing molecules (Fig. 6A). To further evaluate whether downstream signalling components of the type I IFN pathway were affected, both total and phosphorylated forms of IKKε, TBK1, and IRF3 were analysed (Fig. 6B). Similar to the upstream sensors, the expression of these proteins remained unchanged in response to CoCl₂, and their phosphorylation status was undetectable, suggesting the absence of the IFN canonical pathway activation. To delineate the underlying mechanism, RNAseq log_2_-fold-change analysis revealed significant upregulation of *RIG-I* (∼1.5-fold), *IKBKB* (∼1.5-fold), *p65* (∼1.8-fold), and *TRAF6* (∼2.3-fold) (Fig. 6C, Supplementary Table S1). All together, these findings indicate that *A3A* induction following CoCl₂ appears to occur through an alternative sensing or stress-response pathway that operates independently of type I IFN signalling.

Given the central role of TRAF6 as a signalling adaptor downstream of RIG-I that bridges innate sensing to NF-κB activation [56, 57], we next investigated whether NF-κB could function as the transcriptional driver of *A3A* expression under hypoxic stress. NF-κB is a central transcription factor that orchestrates inflammation, immune responses, cell survival, and stress signalling. Its activation is triggered by the phosphorylation of IκBα by the IKK complex, particularly IKKα, leading to IκBα degradation and subsequent nuclear translocation of NF-κB subunits. Consistent with this signalling cascade, CoCl₂ treatment upregulated *IKBKB* expression (Fig. 6C), suggesting that NF-κB activation contributes to *A3A* transcriptional response.

To determine NF-κB involvement in *A3A* induction, we assessed IκBα degradation and p65 nuclear localization. As shown in Fig. 6D, CoCl₂ treatment induced a dose-dependent decrease in cytosolic IκBα protein levels, accompanied by an increase of p65 in the nucleus and a concomitant decrease in the cytosol, consistent with NF-κB activation. Immunofluorescence confirmed this, demonstrating that while p65 remained cytoplasmic under basal conditions, it translocated to the nucleus upon exposure to 200 or 400 µM CoCl₂ (Fig. 6E).

Together, these findings support a mechanistic model in which mitochondrial stress triggers mtDNA release into the cytosol, where it is transcribed by RNA Pol III into immunostimulatory RNAs. These RNAs bind to RIG-I, leading to TRAF6-mediated activation of NF-κB, which drives IκBα degradation, p65 nuclear translocation, and subsequent induction of *A3A* transcription. Importantly, this pathway bypasses the canonical cGAS/STING axis, unveiling a distinct RNA Pol III-dependent mechanism linking mitochondrial stress directly to *A3A* expression.

## Discussion

Hypoxia, a hallmark of various pathological conditions such as inflammation, cancer, and viral infection, is a potent driver of cellular stress responses that reprogram gene expression to ensure survival. Understanding how cells respond to environmental stress is crucial for elucidating the mechanisms underlying immune activation, genome integrity, and disease progression. In this study, we investigated how hypoxia-like stress, modelled by cobalt chloride treatment, leads to the induction of *A3A*, a cytidine deaminase implicated in both antiviral defense and cancer mutagenesis [58–64]. Our findings revealed a novel pathway linking mitochondrial dysfunction to *A3A* induction, independent of canonical IFN signalling, and mediated instead by RNA pol III/RIG-I/TRAF6/NF-κB. This pathway involves mitochondrial dysfunction, cytosolic release of mtDNA, its subsequent transcription by RNA pol III into immunostimulatory RNA species, and activation of NF-κB signalling. Together, these events lead to robust *A3A* expression and accumulation of DSBs, positioning A3A as a critical effector linking metabolic stress to genome instability and cancer. While THP-1 cells were used as a model system, *A3A* induction has been shown to occur more broadly within the myeloid lineage, indicating that the mechanisms described in this study are not limited to a single *in vitro* model [62, 65, 66].

Several aspects of this model resonate with and build upon previous findings. A3A has long been known as an IFN-stimulated gene, with type I IFN responses (particularly IFNα and IFNβ) serving as major transcriptional activators through STAT-dependent pathways. CoCl₂-induced *A3A* expression occurred independently of detectable type I IFN production, suggesting that alternative, stress-responsive mechanisms are involved. This aligns with studies reporting IFN-independent induction of *A3A* in response to genotoxic agents or oxidative stress [35].

The independence of *A3A* induction from type I IFN signalling is both unexpected and informative, given the well-established overlap between hypoxic stress responses and IFN-regulated transcriptional programs. Our data demonstrate that hypoxia-like stress can engage innate immune and stress-adaptive pathways that converge on *A3A* expression while bypassing canonical IFN/STAT signalling. This highlights a context-dependent mode of A3A regulation, in which metabolic and mitochondrial stress directly activate inflammatory transcriptional circuits, notably NF-κB. Such IFN-independent activation provides a mechanistic explanation for *A3A* expression in hypoxic environments where sustained type I IFN signalling is absent or actively suppressed, particularly within the tumour microenvironment.

Mitochondria are increasingly recognized as central regulators of innate immune signalling, largely through the release of mtDNA under conditions of stress. Studies have shown that mitochondrial dysfunction, whether due to hypoxia, ROS, or chemical agents, promotes the leakage of oxidized mtDNA into the cytosol, where it is sensed by pattern recognition receptors [19–23]. In line with this, genes involved in hypoxia (Fig. 3C), mitochondrial metabolism (Fig. 3D), and inflammatory responses (Fig. 3E) were significantly enriched, reflecting an active remodelling of mitochondrial networks to adapt to hypoxic stress. In parallel, pathways related to oxidative stress, DNA repair, and apoptosis were also modulated, suggesting a coordinated cellular strategy to preserve homeostasis under adverse conditions. These transcriptomic changes underscore the intricate crosstalk between energy metabolism and adaptive signalling during hypoxia, positioning mitochondrial reprogramming as a key nexus linking metabolic stress, genomic maintenance, and innate immune activation. This transcriptional landscape is emblematic of the metabolic shift long described in hypoxic cancer cells, known as the *Warburg effect*, wherein cells suppress mitochondrial respiration in favour of enhancing glycolytic flux, alongside activation of stress response programs.

Critically, our findings establish cytosolic mtDNA as an upstream driver of *A3A* activation. DNase I-expressing THP-1 cells exhibited markedly reduced *A3A* expression and DNA damage upon hypoxic stress (Fig. 5A and B), directly implicating mtDNA release as the initiating signal. In the same vein, mtDNA release has been implicated in various inflammatory processes associated with autoimmune diseases and neurodegeneration. In autoimmune diseases, such as systemic lupus erythematosus, mtDNA release can trigger the cGAS/STING pathway, leading to the production of type I IFNs and pro-inflammatory cytokines [67]. Similarly, in neurodegenerative diseases like Alzheimer’s and Parkinson’s, mtDNA leakage contributes to neuroinflammation. Oxidized mtDNA fragments can activate the NLRP3 inflammasome and other pro-inflammatory pathways, exacerbating neuronal damage [68]. However, our work extends this paradigm by linking mtDNA release to a mutagenic pathway with direct consequences for genome integrity, thereby defining mitochondrial stress as a critical molecular crossroads between innate immune signalling and genomic instability.

We further delineate the signalling cascade linking mtDNA release to *A3A* induction. Although cGAS/STING is the canonical cytosolic DNA-sensing pathway, both pharmacological inhibition and transcriptomic profiling revealed that this pathway is dispensable in our system. Instead, our findings identify RNA pol III as a key mediator, consistent with its capacity to transcribe cytosolic mtDNA into 5′-ppp RNA that subsequently activates RIG-I [19, 31–33]. Inhibition or siRNA-mediated knockdown of RNA Pol III subunits markedly reduced *A3A* expression, confirming its central role in this pathway. Upregulation of *RIG-I, TRAF6, IKBKB*, and *p65*, together with IκBα degradation and p65 nuclear translocation, strongly supports activation of the RIG-I/TRAF6/NF-κB pathway. This provides a direct transcriptional mechanism for *A3A* induction under hypoxia, bypassing IFN signalling.

NF-κB is a well-established regulator of inflammatory and survival genes, with p65 shown to directly activate the *A3A* promoter [35]. In our study, CoCl₂ treatment triggered IκBα degradation and P65 nuclear accumulation, hallmarks of canonical NF-κB activation. Immunofluorescence confirmed p65 translocation, and inhibition of upstream activators such as RNA Pol III attenuated both p65 activity and *A3A* expression. Together, these results reinforce and mechanistically contextualize the contribution of NF-κB signalling to stress-induced *A3A* regulation.

Hypoxia is a hallmark of the tumour microenvironment and plays a central role in tumour progression, therapy resistance, and immune evasion. Multiple cancers, including breast, lung, and pancreatic tumours, exhibit regions of severe oxygen deprivation, characterized by mitochondrial dysfunction and increased ROS production [69–71]. Within this context, our findings provide a mechanistic explanation for how hypoxia can contribute to tumour mutagenesis through *A3A* activation. Our data suggest that hypoxic stress directly promotes *A3A* activation in an IFN-independent manner, thereby fuelling tumour evolution. This mechanism may help account for the widespread presence of A3A mutational signatures in cancers with limited evidence of chronic IFN signalling. By linking mitochondrial dysfunction to A3A-mediated mutagenesis, our study establishes a mechanistic bridge between metabolic stress in the tumour microenvironment and the genomic plasticity that underlines tumour progression. Notably, *A3A* expression is frequently upregulated across a range of human cancers, and A3A-associated mutations, particularly C-to-T and C-to-G transitions within the TCW motif, constitute a dominant mutational signature in breast, bladder, cervical, and head and neck cancers [58, 61, 72, 73].

Consistent with this model, transcriptomic analyses from *The Cancer Genome Atlas* (TCGA) reveal strong correlations between *A3A* expression and hypoxia-associated gene signatures across multiple tumour types [74]. Thus, A3A activation in the hypoxic tumour niche may therefore serve as a key driver of intratumoural heterogeneity, clonal evolution, and therapy resistance, particularly in immune-rich or myeloid-infiltrated cancer where APOBEC activity is prominent. In this regard, A3A functions as a double-edged sword, contributing to antiviral and antitumour immunity under acute stress, yet promoting mutational burden and tumour adaptation under chronic activation. Beyond cancer, this pathway may also be relevant to other pathologies characterized by mitochondrial stress and cytosolic mtDNA accumulation. Determining whether aberrant *A3A* induction contributes to pathogenesis in these settings, either by exacerbating inflammation or by driving somatic mutations in affected tissues, will be an important area of future investigation

Finally, our study uncovers a novel RNA Pol III/RIG-I/TRAF6/NF-κB signalling axis that connects hypoxia-induced mitochondrial stress to *A3A* induction and consequent DNA damage. This pathway bypasses canonical IFN signalling and directly links metabolic stress to genomic instability. By uncovering a non-canonical route of *A3A* regulation, our study provides new insight into how hypoxic microenvironments can fuel mutagenesis and evolution in cancer and possibly other diseases. Targeting components of this pathway may offer opportunities to mitigate APOBEC3-driven mutational processes and their downstream consequences. Future studies should explore the *in vivo* relevance of this mechanism in hypoxic cancer models and determine whether targeting components of this pathway could limit *A3A*-driven mutagenesis and its oncogenic consequences.

## Supplementary Material

ugag012_Supplemental_Files

## Data Availability

The raw and processed RNA sequencing data supporting the findings of this study are available on Gene Expression Omnibus (GEO) with the following accession number: GSE312974. All the bioinformatics scripts used in this study are provided on Zenodo at https://doi.org/10.5281/zenodo.17830727. The repository has been made to support data reproducibility and open science.

## Notes

Present address: Namur Research Institute for Life Sciences (NARILIS), Integrated Veterinary Research Unit (URVI), University of Namur, Namur, Belgium
