# Supplementary material for: Hypoxia-mimicked mitochondrial stress triggers APOBEC3A-mediated DNA damage via non-canonical innate immune activation
Source: NAR Mol Med. 2026 Feb 3;3(1):ugag012. doi: 10.1093/narmme/ugag012 (PMC12926716; doi:10.1093/narmme/ugag012)
Supplement: ugag012_Supplemental_Files [file ugag012_supplemental_files.zip › Supp. Figure and Table.pdf]

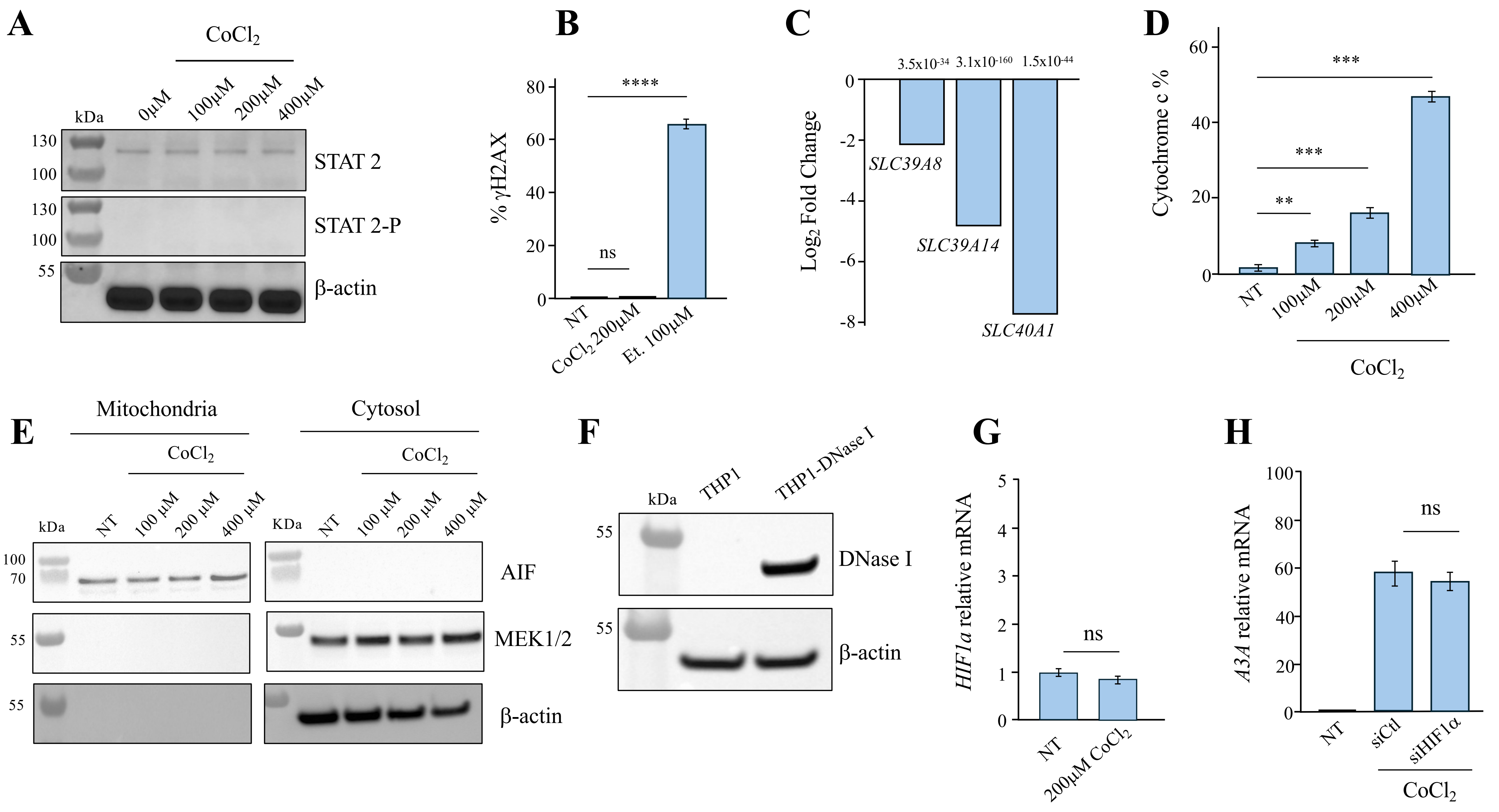

A
CoCl2
0μM
100μM
200μM
400μM
kDa
130
100
STAT 2
130
100
STAT 2-P
55
β-actin
B
% γH2AX
80
60
40
20
0
****
ns
NT
CoCl2 200μM
Et. 100μM
C
Log2 Fold Change
0
-2
-4
-6
-8
3.5x10-34
3.1x10-160
1.5x10-44
SLC39A8
SLC39A14
SLC40A1
D
Cytochrome c %
60
40
20
0
***
***
**
NT
100μM
200μM
400μM
CoCl2
E
Mitochondria
Cytosol
CoCl2
CoCl2
kDa
NT
100 μM
200 μM
400 μM
KDa
NT
100 μM
200 μM
400 μM
100
70
AIF
55
MEK1/2
55
β-actin
F
kDa
THP1
THP1-DNase I
55
DNase I
55
β-actin
G
HIF1a relative mRNA
5
4
3
2
1
0
ns
NT
200μM CoCl2
H
A3A relative mRNA
100
80
60
40
20
0
ns
NT
siCtl
siHIF1α
CoCl2

Supplementary Figure 1

Supplementary Figure 1Effects of $CoCl_2$ treatment on DNA damage response, gene expression, and mitochondrial function in THP-1 and QT6 cells.

A) Western blot analysis of STAT2 and phospho-STAT2 at 20 hours post-treatment of THP-1 cells with 100μM, 200μM, or 400μM $CoCl_2$, compared with untreated cells. β-actin was used as a loading control. B) Flow cytometry analysis of γH2AX-positive QT6 cells following treatment with 200μM $CoCl_2$, compared with untreated controls and with cells treated with 100μM etoposide as a positive control. C) RNAseq data was extracted from Supplementary Table S1 to show Log_2 fold change expression of *SLC39A8*, *SLC39A14*, and *SLC40A1* following DGE. D) Cytochrome c release from THP-1 cells treated with 100μM, 200μM, and 400μM of $CoCl_2$, compared to untreated control cells. E) Analysis of cellular fractions from THP-1 cells treated with 100μM, 200μM, or 400μM $CoCl_2$, compared with untreated controls. Fraction purity was verified by immunoblotting, β-actin as a cytosolic marker, AIF (apoptosis-inducing factor) for mitochondria, and MEK1/2 as an additional cytosolic marker. Molecular weight is indicated in kDa. F) Western blot verification of the THP-1 stable cell line expressing DNase-I-mCherry, with β-actin used as a loading control. G) *HIF1a* relative expression in 200μM $CoCl_2$-treated THP-1 cells compared with untreated cells (NT). Error bars represent standard deviation from three independent experiments. H) *A3A* relative expression in 200μM $CoCl_2$-treated THP-1 cells transfected with control siRNA or *HIF1α* targeting siRNA, compared with untreated cells (NT). Error bars represent standard deviation from four independent experiments. Data obtained in B, D, G and H were subjected to two-way ANOVA, followed by an ad hoc test, *, $p < 0.05$, **$p < 0.01$, ***, $p < 0.001$, and ****, $p < 0.0001$, ns: not statistically significant.

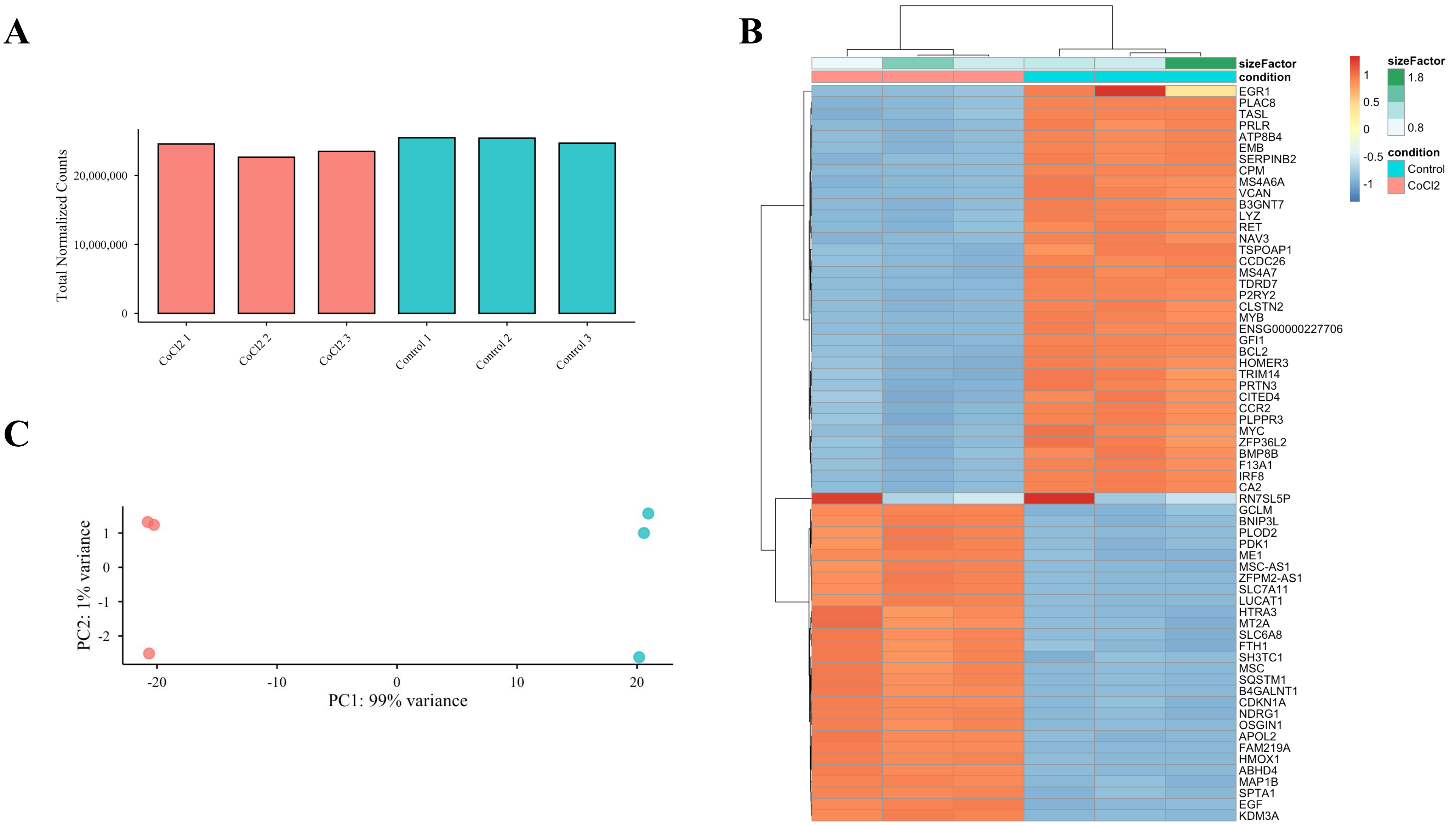
A
Total Normalized Counts
20,000,000
10,000,000
0
CoCl2 1
CoCl2 2
CoCl2 3
Control 1
Control 2
Control 3
B
sizeFactor
condition
EGR1
PLAC8
TASL
PRLR
ATP8B4
EMB
SERPINB2
CPM
MS4A6A
VCAN
B3GNT7
LYZ
RET
NAV3
TSPOAP1
CCDC26
MS4A7
TDRD7
P2RY2
CLSTN2
MYB
ENSG00000227706
GFI1
BCL2
HOMER3
TRIM14
PRTN3
CITED4
CCR2
PLPPR3
MYC
ZFP36L2
BMP8B
F13A1
IRF8
CA2
RN7SL5P
GCLM
BNIP3L
PLOD2
PDK1
ME1
MSC-AS1
ZFPM2-AS1
SLC7A11
LUCAT1
HTRA3
MT2A
SLC6A8
FTH1
SH3TC1
MSC
SQSTM1
B4GALNT1
CDKN1A
NDRG1
OSGIN1
APOL2
FAM219A
HMOX1
ABHD4
MAP1B
SPTA1
EGF
KDM3A
1
0.5
0
-0.5
-1
sizeFactor
1.8
0.8
condition
Control
CoCl2
C
PC2: 1% variance
1
0
-1
-2
-20
-10
0
10
20
PC1: 99% variance

Supplementary Figure S2

Heatmap of gene expression profiles in control and $CoCl_2$-treated cells.

A) Raw read counts were normalized using the means-of-ratios method implemented in DESEQ2. The distribution of normalized counts across samples (n=6) is shown, with control samples in green teal and $CoCl_2$-treated samples in pink. B) Principal Component Analysis (PCA) plot displaying the overall variance in gene expression profiles between the samples. The first principal component (PC1) captures the largest source of variance (99%), separating the control (teal) and $CoCl_2$-treated samples. C) Heatmap of top variable genes (n=65) across all samples, selected based on the highest row variance in the variance-stabilized data (vsd). Gene expression values are scaled by row (z-score). Columns are annotated by treatment type (control in teal and $CoCl_2$-treated in pink), and both genes and samples are hierarchically clustered.

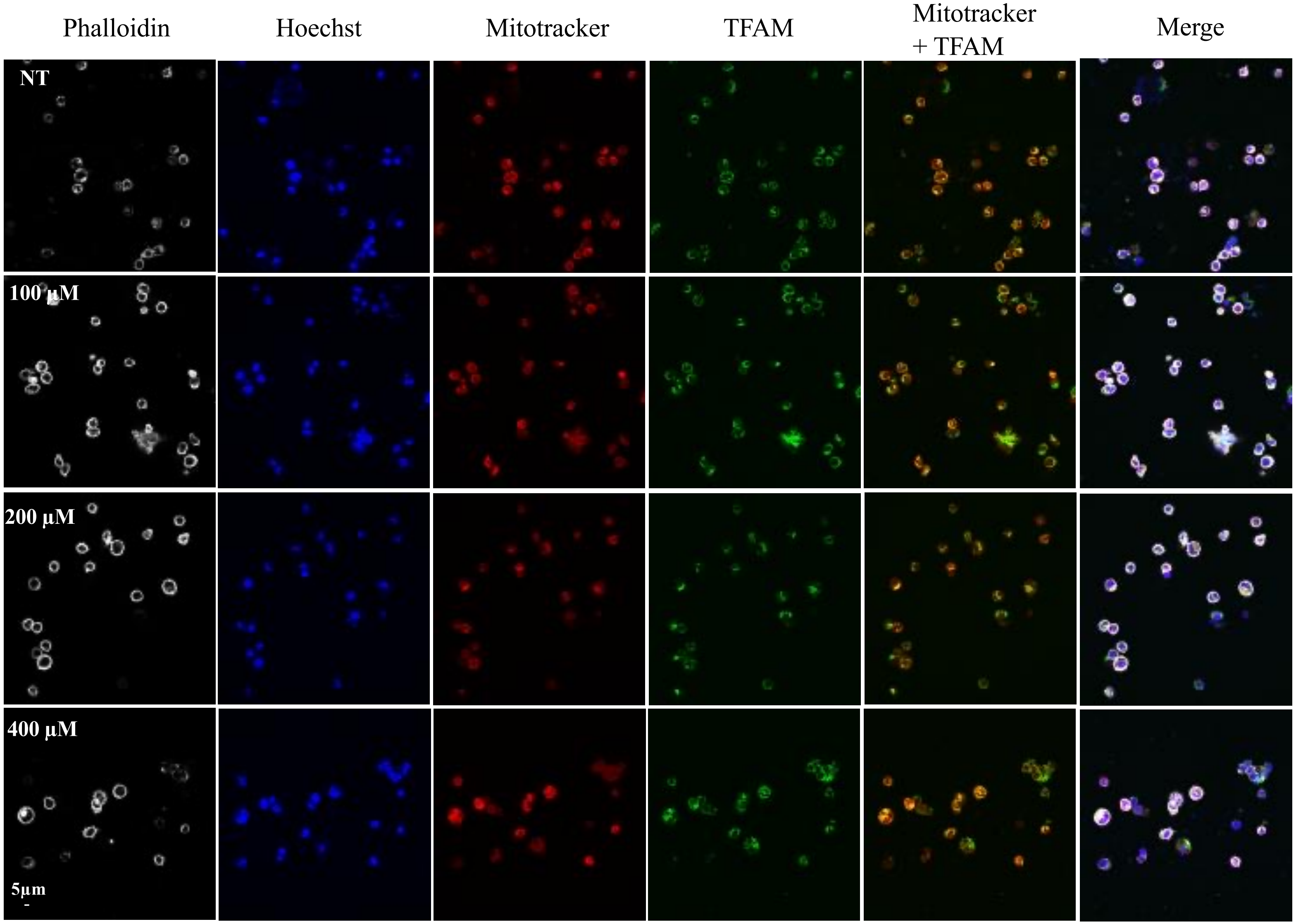

Phalloidin
Hoechst
Mitotracker
TFAM
Mitotracker + TFAM
Merge
NT
100 µM
200 µM
400 µM
5µm

Supplementary Figure S3

Immunofluorescence analysis of $CoCl_2$-Treated THP-1 cells.

Immunofluorescence analysis of THP-1 cells treated with 100μM, 200μM, or 400μM $CoCl_2$ for 20 hours, compared with untreated controls, using confocal microscopy and an increased number of cells. Actin filaments were stained with phalloidin (white), nuclei with Hoechst (blue), mitochondria with MitoTracker (red), and mtDNA with a monoclonal anti-TFAM antibody (green). These larger fields of view were used for mitochondrial network quantification shown in Fig. 4B.

Supplementary Table S1

Complete list of Differentially Expressed Genes (DEG) obtained using the DESEQ2 package in R comparing the $CoCl_2$ treated versus untreated samples submitted for RNAseq.

**Primer**	**Sequence**	**Probe**
IFNα fwd	5’TCCTGCTTGAAGGACAGACA	*UPL63*
IFNα rev	5’TTTCAGCCTTTTGGAACTGG	
IFNβ fwd	5’CTTTGCTATTTTCAGACAAGATTCA	*UPL20*
IFNβ rev	5’GCCAGGAGGTTCTCAACAAT	
MTF3212 fwd	5’CACCCAAGAACAGGGTTTGT	
MTF3319 rev	5’TGGCCATGGGTATGTTGTTAA	
HCoxI fwd	5’CCTCCCTTAGCAGGGAACTAC	
HCoxI rev	5’CACCTGCTAGGTGTAAGGAGAAG	
HIF1α fwd	5’TTTTCAAGCAGTAGGAATTGGAA	
HIF1α rev	5’GTGATGTAGTAGCTGCATGATCG	
β2M fwd	5’AATCAGATGGGTGTAGATCAAGG	
β2M rev	5’GTTTCCACCCCTTCCATTTT	
RPL13 fwd	5’CTGGACCGTCTCAAGGTGTT	*UPL74*
RPL13 rev	5’GCCCCAGATAGGCAAACTT	

Supplementary Table S2

Compendium of primers used for PCR, qPCR and SYBR Green. Fwd: forward, rev: reverse.
